# Recent advances in crocodilian oil research: bioactive components and potential therapeutic applications

**DOI:** 10.3389/fmed.2025.1573925

**Published:** 2025-06-18

**Authors:** Yuxi Huang, Xinyue Zheng, Xingchen Ming, Qiqi Jiao, Weihao Xiao, Qian Wu, Linyang Zheng, Yunfu Zeng, Shaowen Cheng, Rong Wang, Jian Yang, Yangyang Bian, Jiangling Yao

**Affiliations:** ^1^The First Clinical College, Hainan Medical University, Haikou, China; ^2^School of Basic Medicine and Life Sciences, Hainan Medical University, Haikou, China; ^3^The Second Clinical College, Hainan Medical University, Haikou, China; ^4^Emblem of the Emergency and Trauma College, Hainan Medical University, Haikou, China; ^5^Key Laboratory of Emergency and Trauma of Ministry of Education, Wound Repair Department, Key Laboratory of Hainan Trauma and Disaster Rescue, The First Affiliated Hospital of Hainan Medical University, Haikou, China

**Keywords:** crocodilian oil, anti-inflammatory, skin health, energy metabolism, antimicrobial activity, scar reduction

## Abstract

The economic value of crocodilian farming has risen substantially in recent years, drawing increasing attention to crocodilian oil as a traditional natural remedy rich in diverse bioactive constituents. Despite its therapeutic potential, crocodilian fat remains underutilized, and its nutritional and medicinal properties have not been widely recognized. This review provides a bibliometric analysis of past research trends and highlights current developments related to crocodilian oil. Recent advances in the characterization of its physicochemical properties and health-related applications are summarized. The primary biological activities of this oil are attributed to its high unsaturated fatty acid and stearic acid contents. Emerging evidence supports its anti-inflammatory, antimicrobial, and scar-reducing effects mediated through key signaling pathways, including p38 mitogen-activated protein kinase, transforming growth factor-β1/Smad3, and AMP-activated protein kinase. Reported benefits include improvements in skin conditions and the modulation of energy metabolism. Potential applications encompass adjunctive treatment for *Candida albicans* infections, topical anti-inflammatory agents, moisturizers, and permeability enhancers in cosmetic formulations, and dietary oil substitutes for managing hypertriglyceridaemia and metabolic disorders affecting the liver and brain. Challenges and future research directions in this field are also discussed.

## 1 Introduction

Having existed for approximately 85 million years, crocodilians are often referred to as “living fossils.” Their use in traditional Chinese medicine dates back to the 16th-century Ming Dynasty (1). Historically, the crocodilian industry has prioritized skin processing, leaving by-products such as meat, fat, bone, and blood—comprising nearly 90% of the animal’s mass—largely underutilized and primarily consumed as food. Beyond their nutritional value, these by-products hold medicinal potential and their further processing has been proposed to enhance their utility. Increasingly, crocodilian-derived materials are being explored for applications in medicine, food, and chemical industries (2).

Crocodilian oil, extracted from adipose tissue, has long been used as a traditional remedy. Current studies on crocodilian oil predominantly utilize raw materials sourced from captive-bred crocodilians. The Compendium of Materia Medica documented its use for moxibustion-induced sores and other skin conditions (3). Contemporary studies have validated its therapeutic effects, including for frostbite, burns, and microbial infections. These activities are largely attributed to key lipid components, particularly stearic acid and various unsaturated fatty acids. Stearic acid exhibits antimicrobial activity against Gram-positive and Gram-negative bacteria, including multidrug-resistant *Staphylococcus epidermidis* and vancomycin-resistant *Enterococcus faecalis*, especially when delivered via liposomes. However, the antibacterial efficacy of palmitic acid remains inconsistent (4).

Crocodilian oil is rich in omega-3, omega-6, and omega-9 fatty acids, each contributing to its bioactivity. Omega-3 fatty acids have anti-inflammatory and immunomodulatory properties, and promote wound healing (5, 6). Linoleic acid, the dominant omega-6 in crocodilian oil, is essential for skin barrier integrity; its deficiency impairs skin function and increases transepidermal water loss (7). Omega-9 fatty acids, such as oleic acid, activate sirtuin 1 (SIRT1), a NAD^+^-dependent deacetylase that regulates inflammation and oxidative stress (8). Crocodilian oil may also activate peroxisome proliferator-activated receptor gamma (PPAR-*γ*), contributing to anti-inflammatory effects, improved cellular survival, and reduced neutrophil migration—actions believed to be PPAR-γ dependent (9).

**Figure 1:**
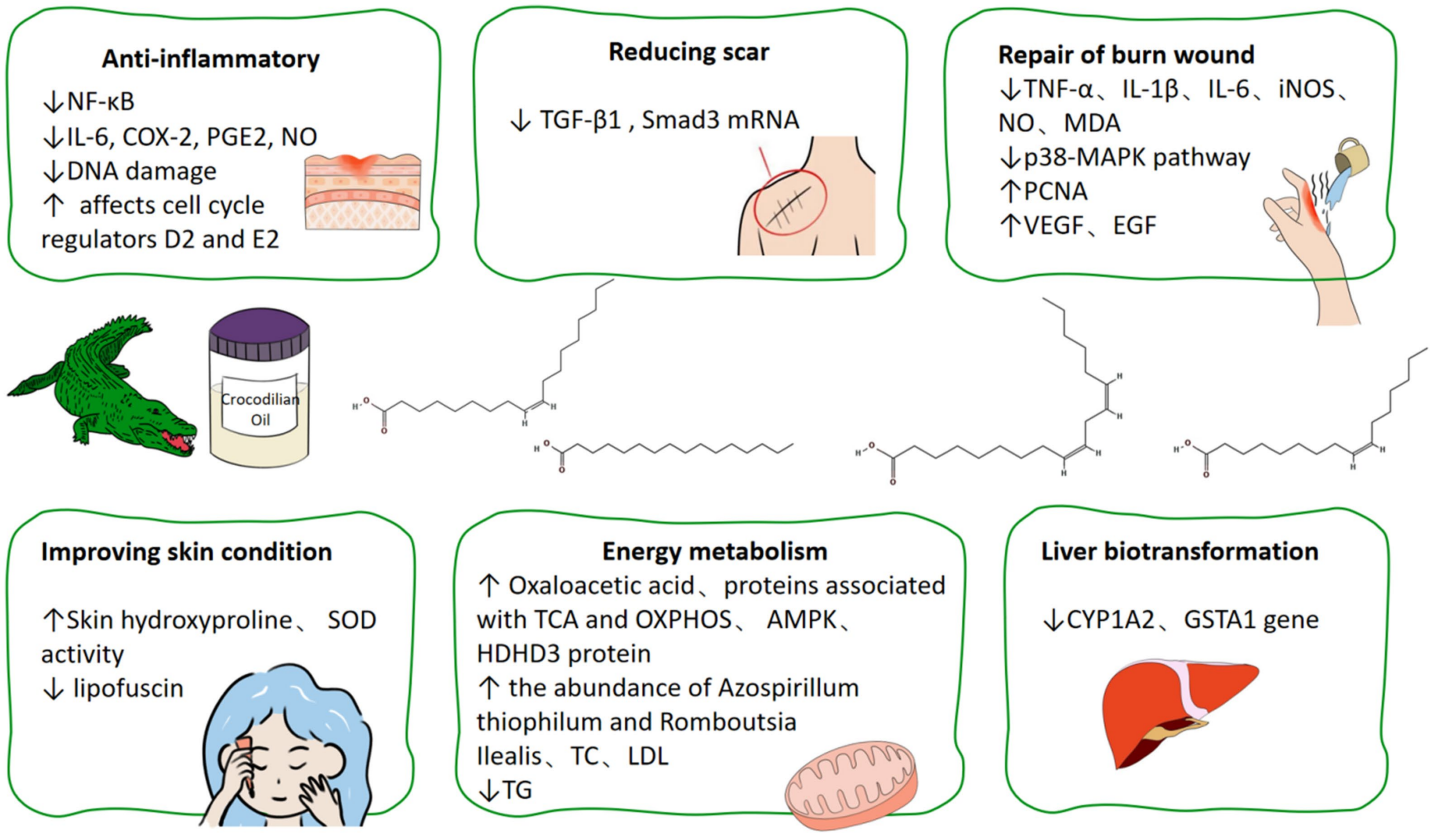
Schematic overview of the review theme and key topics covered, including the biological effects and chemical composition of crocodilian oil. Crocodilian oil exhibits six primary pharmacological actions: anti-inflammatory, scar reduction, burn wound healing, skin improvement, energy metabolism regulation, and hepatic biotransformation. Bioactive components include high unsaturated fatty acids and stearic acid. Emerging evidence supports its anti-inflammatory and antimicrobial effects mediated through p38 mitogen-activated protein kinase (MAPK), transforming growth factor-β1/Smad3, and AMP-activated protein kinase (AMPK) pathways.

This review summarizes the physicochemical properties and therapeutic potential of crocodilian oil, offering a scientific basis for its application in the food, chemical, and medical sectors. Figure 1 outlines the scope of the review and the associated biological effects of crocodilian oil.

## 2 Bibliometric analysis

In addition to its recognized therapeutic potential, several challenges and inconsistencies persist in current research on crocodilian oil. While stearic acid and unsaturated fatty acids are consistently reported to possess antimicrobial and anti-inflammatory properties, findings regarding palmitic acid remain contradictory. Some studies indicated notable antimicrobial activity, whereas others reported minimal or no effect (10).

To better understand these discrepancies and evaluate the broader research landscape, a bibliometric analysis was performed. Relevant publications were retrieved from the Web of Science Core Collection, specifically the Science Citation Index Expanded, using the search terms TS = (“crocodile oil” OR “Crocodylus oil” OR “crocodilian oil” OR “Crocodylidae oil” OR “alligator oil” OR “Alligatoridae oil” OR “caiman oil” OR “gharial oil” OR “Gavialis oil”). This search yielded 15 relevant articles, which were exported in plain text format with full records and cited references for further analysis.

**Figure 2:**
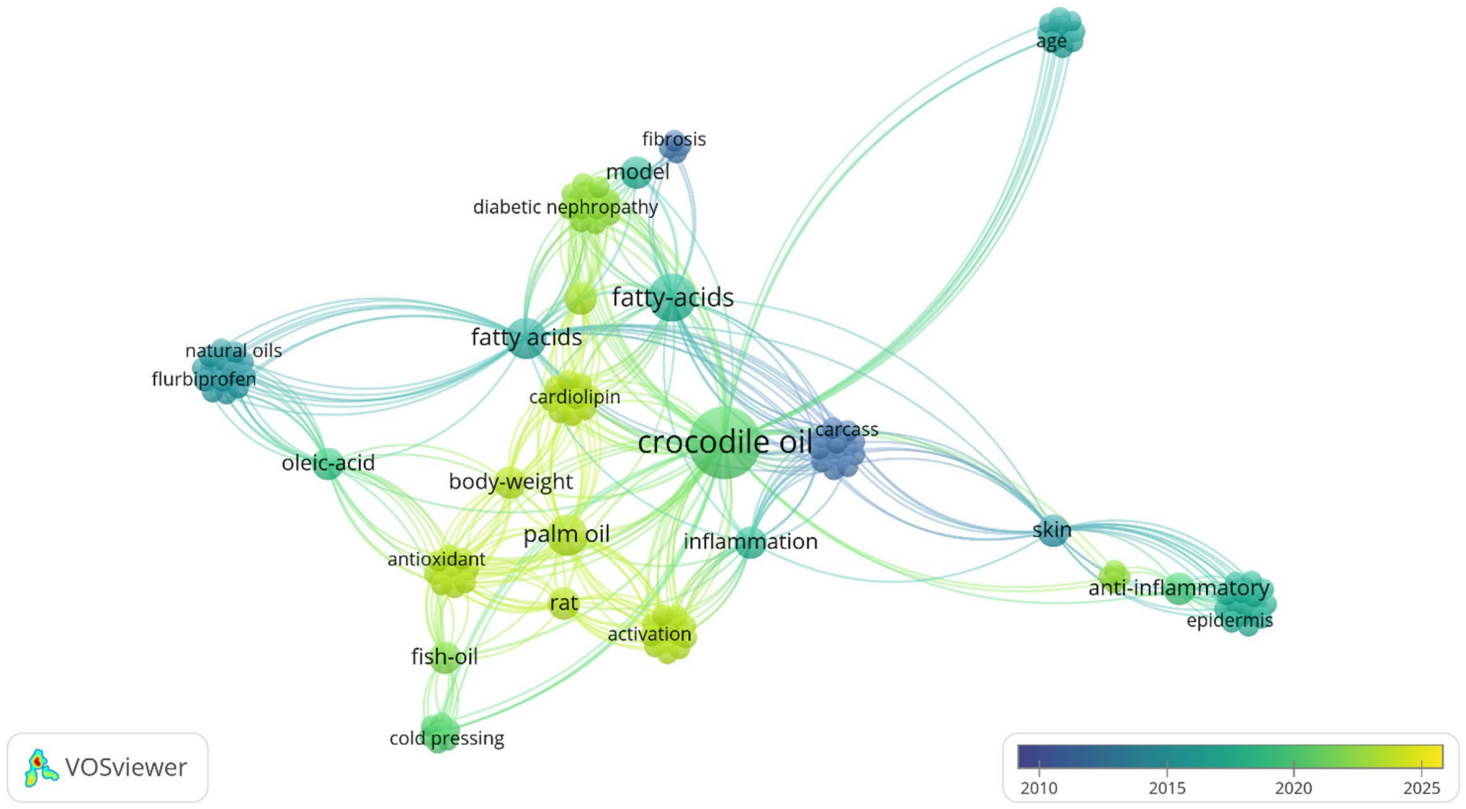
Progression of keywords in crocodilian oil research over time. Co-occurrence network of 157 keywords from the Web of Science Core Collection (2015–2025). High-frequency terms include “fatty acids,” “antioxidants” and “epidermis” reflecting core research foci. Overlay visualization shows shifting priorities, with recent studies (2020–2023, yellow clusters) emphasizing antioxidant properties, palmitic acid effects, and emerging concerns about potential nephrotoxicity in diabetic models. Circle size indicates keyword frequency; connecting lines represent co-occurrence strength.

Network visualizations were generated using VOSviewer (version 1.6.16) and CiteSpace (version 6.2.2), with high-resolution outputs saved in PNG or PDF format. The VOSviewer co-occurrence network (Figure 2) revealed 157 keywords across the selected articles, with the most frequent being “fatty acids,” “antioxidants,” and “epidermis,” reflecting core research themes. Overlay visualization illustrated the temporal evolution of these keywords, indicating a shift in focus. Recent studies have increasingly investigated the antioxidant properties of crocodilian oil and the role of palmitic acid, including concerns about its potential nephrotoxicity in diabetic models—reflecting an expanding interest in its systemic health effects.

**Figure 3:**
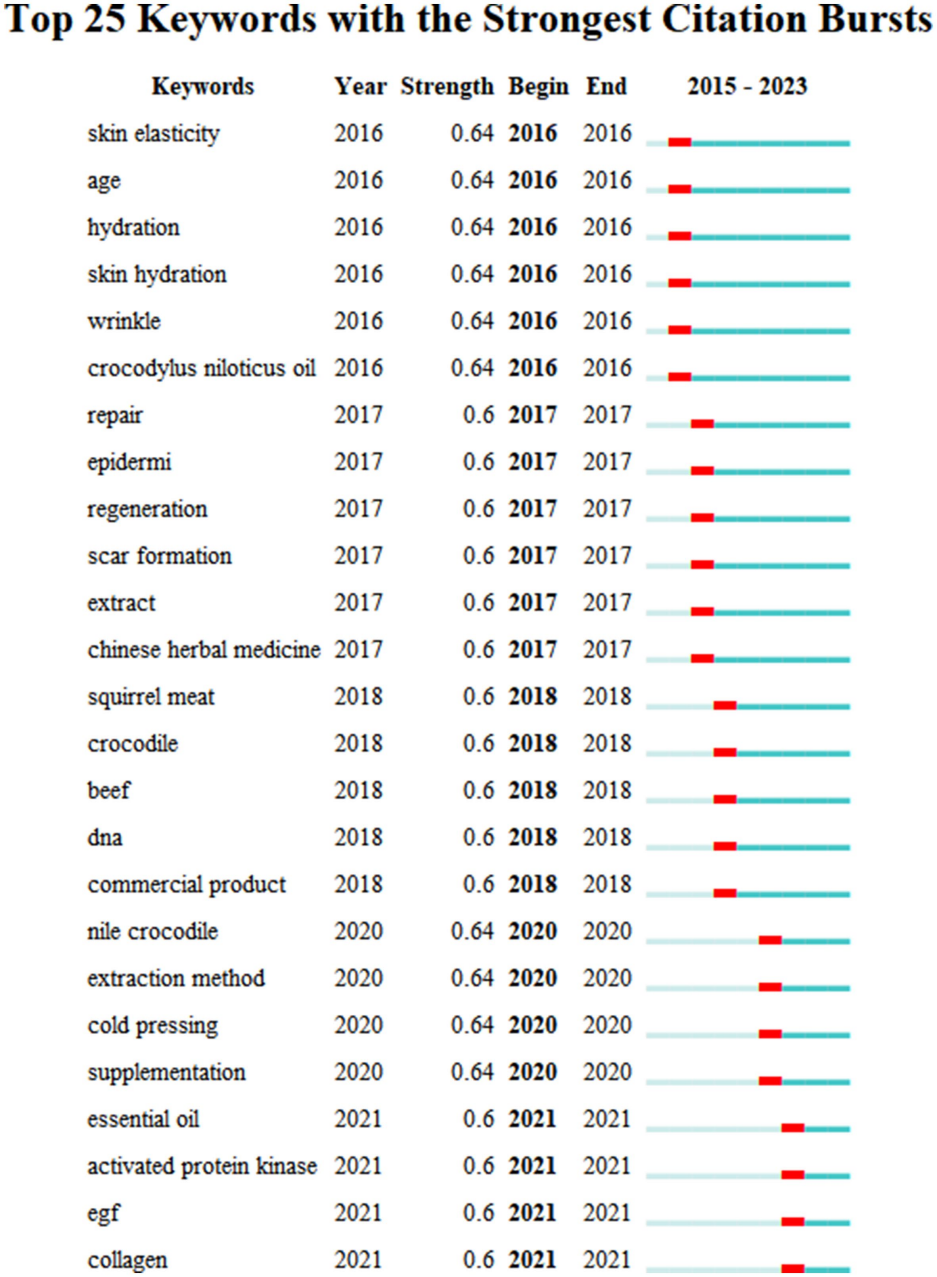
Top 25 keywords with the strongest citation bursts identified by CiteSpace. Analysis reveals three evolutionary phases: Early focus (2016–2018) on cosmetic applications (skin elasticity, hydration, wrinkle reduction); transitional phase (2019–2020) exploring extraction methods and supplements; recent emphasis (2021–2023) on molecular mechanisms involving AMPK, epidermal growth factor (EGF), and collagen biosynthesis.

CiteSpace burst detection analysis (Figure 3) further highlighted dynamic changes in research emphasis from 2015 onwards. Between 2016 and 2018, prominent keywords included skin elasticity, aging, hydration, and wrinkles, indicating an early emphasis on cosmetic and dermatological applications. Since 2020, research has increasingly focused on extraction methods, essential oils, and supplementation, signaling advancements in formulation and processing. Notably, from 2021 onwards, emerging keywords, such as AMP-activated protein kinase (AMPK), epidermal growth factor (EGF), and collagen, point to a growing focus on the molecular mechanisms underlying crocodilian oil’s role in skin regeneration and repair. These trends underscore the need for further investigation of its cellular and molecular interactions.

## 3 Physical and chemical properties

Crocodilian oil is a light-yellow, animal-derived oil with a characteristic fishy odor and has potential as an edible oil. It remains liquid at room temperature and solidifies at 4°C. Its relative density at 22°C ± 5°C is 0.8983 g/cm^3^. Key physicochemical parameters include an iodine value of 99 g/100 g, acid value of 0.2685 mg/g, saponification value of 191 mg/g, peroxide value of 33.73 meq/kg, and viscosity of 12 mPa·s at 30°C. It is insoluble in water and acidic solvents but dissolves readily in organic solvents and strong alkaline solutions, with optimal solubility in diethyl ether and petroleum ether (11).

**Figure 4:**
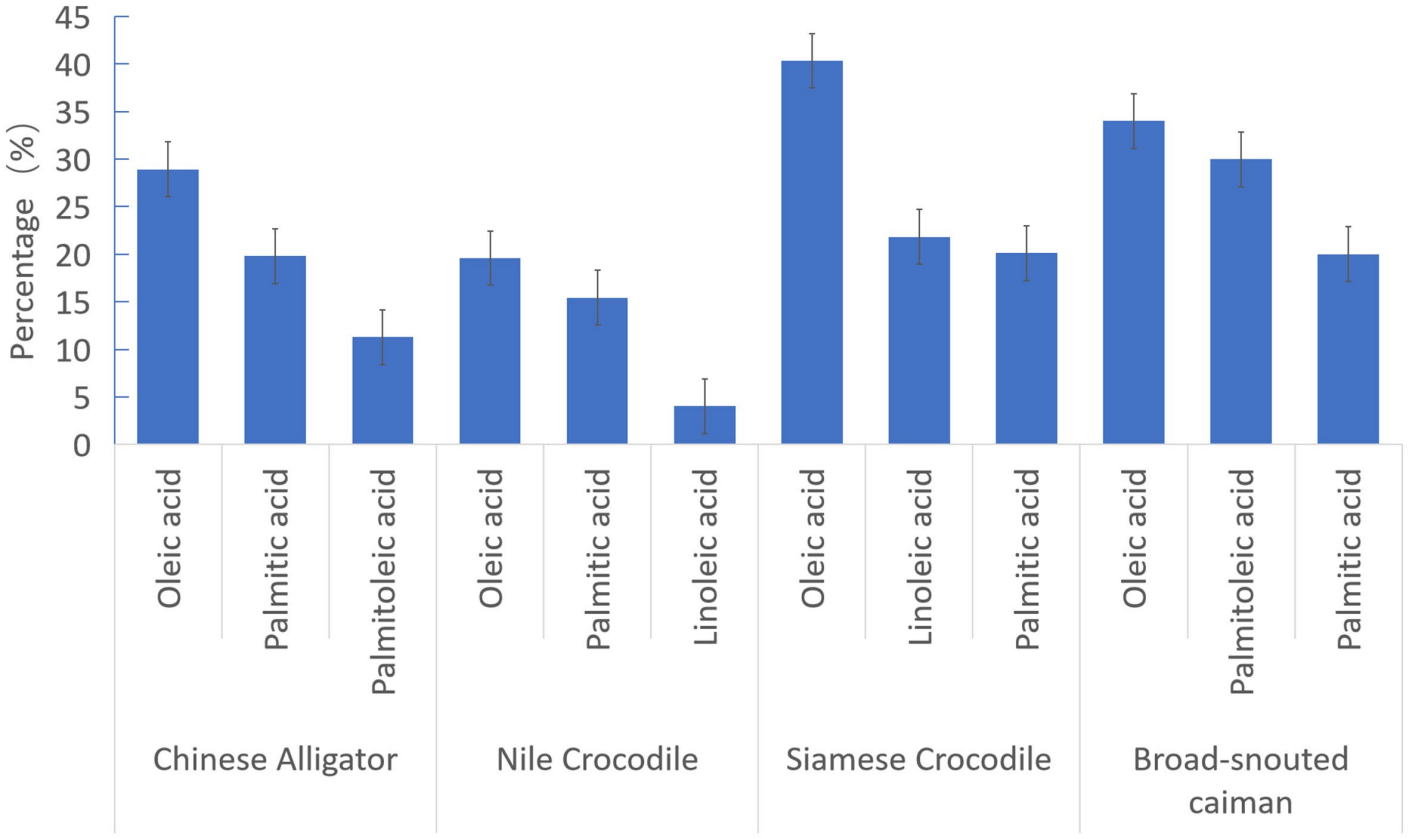
Percentage composition of major fatty acids in crocodilian oil. Percentage distribution of primary fatty acids in crocodilian oil across four species (Chinese Alligator, Nile Crocodile, Siamese Crocodile and Broad-snouted caiman), highlighting oleic acid as the predominant component (up to 40%), followed by palmitic, linoleic, and palmitoleic acids, with significant variations observed among species.

Crocodilian oils (e.g., crocodile, caiman) typically contain over 80% crude fat content in storage fat, with reported values ranging from 82 to 89% depending on species and extraction methods (2, 12, 13). As shown in Figure 4 (11), the number and proportion of identified fatty acids vary across crocodilian species. Sixteen types of fatty acids have been reported in Nile Crocodile (*Crocodylus niloticus*) oil (14), with oleic acid, palmitic acid and linoleic acid accounting for 19.59, 15.44 and 4.03%, respectively. Twenty-four in Chinese Alligator (*Alligator sinensis*) oil (approximately 71% unsaturated fatty acids) (12), with oleic acid, palmitic acid and palmitoleic acid accounting for 28.94, 19.81 and 11.29%, respectively. The Broad-snouted caiman (*Caiman latirostris*) oil (approximately 74% unsaturated fatty acids) has oleic acid, linoleic acid and palmitic acid accounting for 34, 30 and 20%, respectively, (13). Thirty-two in Siamese Crocodile (*Crocodylus siamensis*) oil (approximately 59% unsaturated fatty acids) (15), with oleic acid, linoleic acid and palmitic acid accounting for 40.35, 21.81 and 20.11%, respectively. Notably, omega-6 and omega-9 fatty acid concentrations in Siamese Crocodile oil are about fourfold higher than those in fish oil (16). Despite interspecies variation, the predominant components—oleic acid, palmitic acid, linoleic acid, and palmitoleic acid—are generally consistent across species. In contrast, levels of eicosapentaenoic acid (EPA) and docosahexaenoic acid (DHA) are relatively low (12).

EPA and DHA, essential omega-3 polyunsaturated fatty acids, play key roles in human health (17). At the molecular level, they modulate inflammation-related gene expression by altering membrane phospholipid composition, disrupting lipid raft integrity, inhibiting nuclear factor kappa B (NF-κB), and activating PPAR-*γ*, thereby promoting anti-inflammatory mediators and suppressing inflammation (18). DHA is highly enriched in neuronal membranes and the brain’s gray matter, where it supports neural transmission, membrane stability, synaptic plasticity, and intercellular signaling (17). Both EPA and DHA contribute to cardiovascular health by lowering triglycerides, regulating blood pressure, enhancing cardiac function, and reducing thrombosis risk (19, 20). Although their specific roles in crocodilian oil remain to be clarified, current evidence suggests involvement in wound healing and tissue regeneration (21). Species differences, environmental conditions, and extraction methods can influence the oil’s composition (22). Its fatty acid profile closely resembles that of human sebum. Oleic acid, in particular, facilitates transdermal drug delivery due to its excellent skin penetration. Additionally, the absence of phospholipids enhances dermal permeability, contributing to improved biocompatibility and absorption in topical applications (23).

Refined crocodilian oil contains trace amounts of erucic acid. Essential metallic elements, in descending order of concentration, include calcium, sodium, potassium, iron, magnesium, zinc, and copper. Heavy metal levels are well below national food safety thresholds. The cholesterol content is 11.34 ± 0.07 μg/100 g in crude oil and decreases to 5.22 ± 0.66 μg/100 g after refining (11).

## 4 Nutritional and medicinal value

Table 1 summarizes the biological functions of various crocodilian oils. Of these, Siamese Crocodile oil has been the most extensively studied, likely due to easier sample availability and broader investigation of its pharmacological mechanisms. Compared to Nile Crocodile and Chinese Alligator oils, studies on Siamese Crocodile oil are supported by more robust experimental evidence and encompass a wider range of biomedical applications, including anti-inflammatory effects, tissue repair, and metabolic regulation.

**Table 1:** Documented biological functions of different types of crocodilian oil.

No.	Type of crocodilian oil	Documented function(s)
1	Nile Crocodile	Antimicrobial (14), improvement in skin condition (40)
2	Siamese Crocodile	Anti-inflammatory (9); scar reduction (33); burn wound healing (30, 34–36); improvement in skin condition (11, 42); modulation of energy metabolism (16, 44–48); effects on liver biotransformation (52); radical scavenging activity (11, 54)
3	Chinese Alligator	Treatment of chilblains (37)

In contrast, research on Nile Crocodile and Chinese alligator oils remains limited, with most studies focused on antibacterial activity, skin barrier function enhancement, and frostbite treatment. Data on oils from other crocodilians are currently lacking.

### 4.1 Wound repair

#### 4.1.1 Antimicrobial properties

The global rise of antibiotic resistance, driven by the prolonged and widespread use of antimicrobial agents, presents a serious public health challenge (24). In response, there is increasing interest in traditional substances with intrinsic antimicrobial activity, such as crocodilian oil, as potential alternatives or adjuncts to conventional antibiotics. Crocodilians inhabit pathogen-rich environments and frequently sustain traumatic injuries during territorial or mating conflicts. Despite these conditions, their wounds often heal rapidly and without infection, suggesting a robust immune system and inherent antimicrobial defenses (14).

Both saturated and unsaturated fatty acids in crocodilian oil have been associated with antifungal and antimicrobial properties (25). Omega-3 fatty acids exhibit broad-spectrum antimicrobial activity (26, 27), while omega-6 fatty acids have shown efficacy against parasitic infections (28). However, the four predominant fatty acids in crocodilian oil—oleic acid, palmitic acid, linoleic acid, and palmitoleic acid—have not demonstrated consistent or strong antimicrobial effects, raising questions about the key contributors to the oil’s observed bioactivity.

*In vitro* studies indicated that crocodilian oil exhibits comparable activity against Gram-positive *Staphylococcus aureus* and Gram-negative *Klebsiella pneumoniae*, suggesting that bacterial cell wall structure does not significantly affect its antimicrobial efficacy. Notably, crocodilian oil demonstrates selective antifungal activity against *Candida albicans* (14), indicating potential therapeutic use in fungal infections. However, research on its anti-infective mechanisms remains limited, and clinical studies evaluating its efficacy against a broader range of pathogens are still lacking.

#### 4.1.2 Anti-inflammatory properties

Inflammation is a complex physiological process involving the activation and release of various mediators, including histamine, prostaglandins, interleukins, lymphokines, and platelet-activating factors (29).

*In vitro* studies by Ngernjan et al. demonstrated that crocodilian oil significantly reduced both synthesis and gene expression of interleukin-6 (IL-6), and inhibited the expression of key pro-inflammatory mediators, including cyclooxygenase-2, prostaglandin E2, nitric oxide (NO), and NF-κB, in lipopolysaccharide-stimulated RAW 264.7 macrophages. Additionally, crocodilian oil attenuated DNA damage and upregulated the expression of cell-cycle regulators, D2 and E2, indicating a role in modulating immune responses (30).

These findings suggest that crocodilian oil may regulate cytokine secretion, particularly IL-6 and tumor necrosis factor-alpha (TNF-*α*), thereby influencing immune cell function. This immunomodulatory potential could help to suppress excessive inflammation or promote immune cell proliferation. However, further studies are needed to clarify its direct effects on specific immune cell populations such as T lymphocytes and macrophages. Current evidence supports its potential use as a nutritional supplement for the prevention of inflammatory disease (9).

Pharmacodynamic studies comparing administration routes showed that oral delivery of crocodilian oil reached peak anti-inflammatory activity at 3 h (60.8% ± 5.5%), whereas topical application peaked at 12 h (57.9% ± 5.9%) (14). While topical use resulted in a more rapid onset and sustained effect, oral administration produced a shorter duration of action. These findings suggest topical application may be more effective for managing inflammatory conditions.

#### 4.1.3 Reduces scar formation

Abnormal scar formation is strongly associated with chronic inflammation and impaired wound healing. These scars, including hypertrophic and keloid types, are often accompanied by pain, pruritus, local dysfunction, and esthetic concerns. A central mechanism driving such pathological scarring is the persistent activation of the transforming growth factor-beta (TGF-*β*)/Smad signaling pathway, which continuously stimulates fibroblasts and myofibroblasts, resulting in excessive collagen deposition and fibrotic remodeling (31, 32).

**Figure 5:**
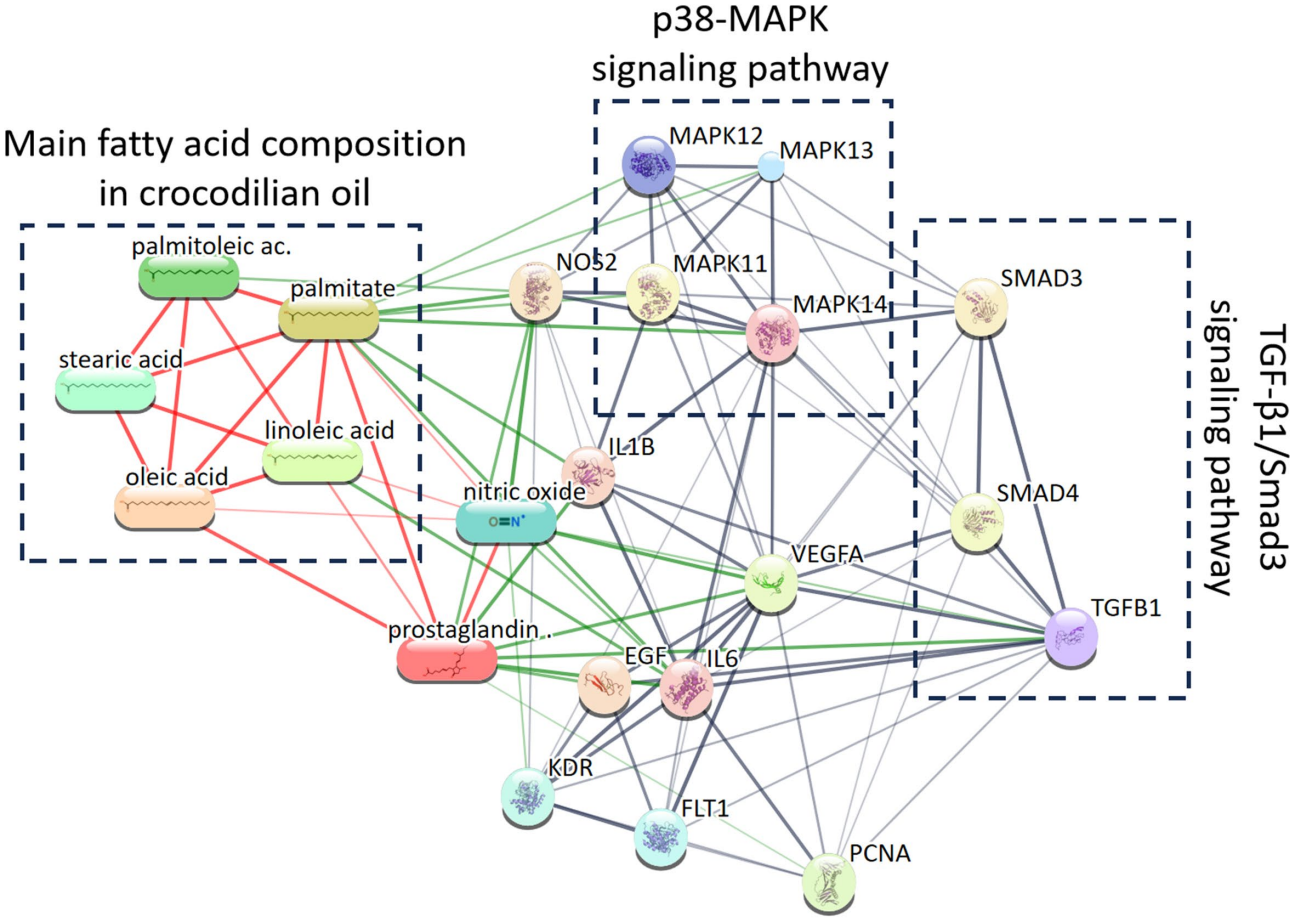
Interaction network of the five principal fatty acids in crocodilian oil with the p38 MAPK and TGF-β1/Smad3 signaling pathways, generated using STITCH v5.0. This figure illustrates the interaction network between key fatty acids in crocodilian oil (including palmitoleic acid, palmitic acid, stearic acid, oleic acid, and linoleic acid) and the p38 MAPK (MAPK12/13/11/14) and TGF-β1/Smad3 (TGFB1, SMAD3/4) signaling pathways, generated using the STITCH database. This study pioneers the systematic mapping of synergistic interactions between crocodilian oil components and growth factors, providing a multi-target framework for understanding their anti-inflammatory and pro-regenerative effects, and advancing the application of natural products in precision medicine.

In a Wistar rat model of deep second-degree burns, Li et al. (33) reported that topical application of crocodilian oil significantly accelerated wound healing, reduced scar formation, and downregulated TGF-β1 and mRNA expression in skin tissues. These results suggest that crocodilian oil may exert antifibrotic effects by modulating the TGF-β1/Smad3 pathway, thereby improving scar outcomes (see Figure 5). Although crocodilian oil–based scar-reducing products are commercially available, their adoption in markets such as China remains limited. Further research is needed to validate their clinical efficacy and support the development of optimized formulations for broader therapeutic use (33).

### 4.2 Burn wound repair

Burn injuries, typically resulting from thermal exposure, cause significant skin damage and initiate a complex healing cascade comprising four overlapping phases: coagulation and hemostasis, inflammation, tissue proliferation and remodeling, and scar formation (34).

In a deep second-degree burn rat model, Hualiang et al. reported that crocodilian oil significantly reduced post-injury levels of pro-inflammatory cytokines, including TNF-*α*, interleukin-1β (IL-1β), and IL-6. It also suppressed phosphorylation of p38 mitogen-activated protein kinase (p38 MAPK), thereby inhibiting downstream signaling and the secondary expression of IL-1β and IL-6. Additionally, crocodilian oil downregulated inducible NO synthase (iNOS), decreasing NO synthesis. These effects collectively mitigated vasodilation, plasma extravasation, oedema, and tissue damage caused by ischemia and hypoxia (30, 35).

Crocodilian oil also upregulated proliferating cell nuclear antigen and increased expression of vascular endothelial growth factor and EGF, promoting fibroblast and endothelial cell proliferation, differentiation, and migration. These processes enhanced collagen synthesis and extracellular matrix deposition, facilitating the transition into the proliferative phase of wound healing.

In a related study, Hualiang et al. developed a crocodilian oil burn ointment, which demonstrated anti-inflammatory, wound-healing, and analgesic effects in deep second-degree burn models. Histological analysis revealed more uniformly distributed hair follicles in treated skin, with higher counts of total, active, primary, and secondary follicles compared to both untreated controls and a silver sulfadiazine treatment group (36). Similarly, Xiang et al. (34) found that crocodilian oil reduced malondialdehyde and TNF-*α* levels, accelerating repair in superficial second-degree burns.

Clinical observations by Yangjian et al. suggested additional benefits of crocodilian oil, including nutritional support, immunomodulation, and enhanced skin integrity in frostbite cases. The use of cooling agents, such as ice tablets, reportedly facilitated subcutaneous absorption and improved therapeutic outcomes (37). Further pharmacodynamic studies have indicated potential applications in acne treatment, anti-aging, and scar improvement, supporting its broader use in dermatology (38).

Despite encouraging results in rodent models, the wound-healing efficacy of crocodilian oil in humans remains underexplored. Limited data exist regarding optimal formulation, dosage, and delivery methods. Additional clinical and pharmacokinetic studies are necessary to validate its therapeutic potential and guide evidence-based use (39).

**Figure 6:**
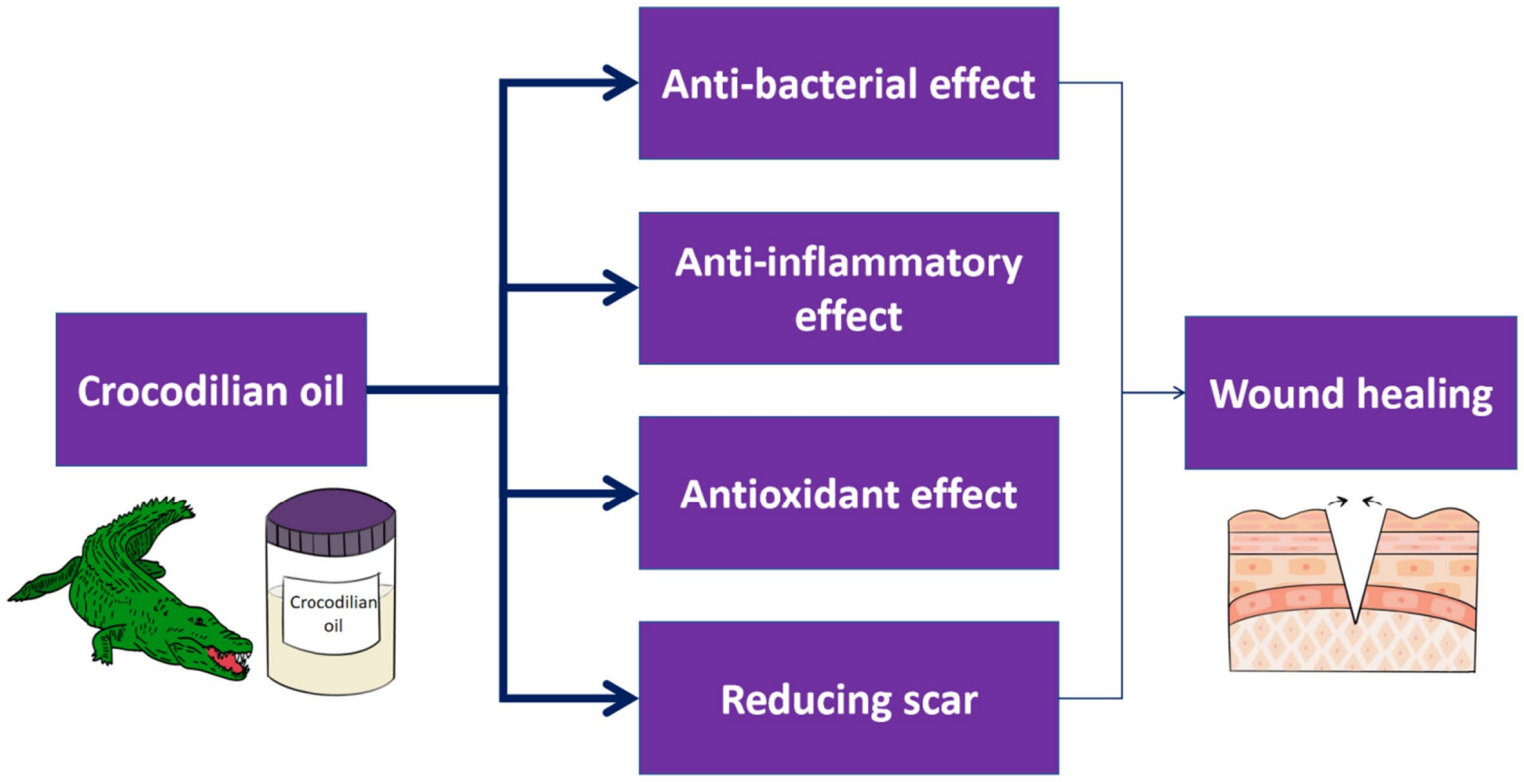
Schematic representation of the biological mechanisms by which crocodilian oil promotes wound healing. Schematic illustration of the biological mechanisms of crocodilian oil in wound repair, highlighting its anti-bacterial, anti-inflammatory, and antioxidant properties, as well as its capacity to reduce scar formation, collectively contributing to accelerated and enhanced wound healing processes.

A summary of the proposed mechanisms by which crocodilian oil promotes wound healing is illustrated in Figure 6.

## 5 Other roles

### 5.1 Improves skin conditions

Crocodilian oil has shown considerable promise in dermatological care, particularly in enhancing skin hydration, elasticity, and barrier function. A study by Venter et al. (40) demonstrated that topical application of Nile Crocodile oil lotion significantly improved skin hydration and elasticity while reducing scaling. These benefits were attributed to the emulsion’s high lipid content, which enhanced emollient properties and more effectively reduced transepidermal water loss compared to formulations with lower lipid concentrations (41).

Crude crocodilian oil also exhibited moisturizing effects comparable to those of glycerol, with superior moisture retention and absorption rates relative to refined oil. Its strong transdermal penetration-enhancing capacity further supports its use as a high-grade moisturizing and permeation-promoting agent in cosmetic formulations. Owing to its lipid composition, which closely resembles human sebum, crocodilian oil demonstrates excellent dermal compatibility, with no reported cases of irritation or allergic reactions. These properties highlight crocodilian oil’s potential for incorporation into transdermal drug delivery systems and advanced skincare products (11).

In an aging mouse model, Chongshu et al. (42) found that crocodilian oil increased hydroxyproline content and superoxide dismutase (SOD) activity while reducing lipofuscin accumulation, suggesting anti-aging effects mediated through collagen support and oxidative stress reduction. Notably, the oil’s efficacy may be influenced by its extraction method. Oils obtained via leaching exhibited lower peroxide values than those from cold pressing, indicating reduced lipid oxidation. Since elevated peroxide levels are associated with the generation of reactive oxygen species (ROS)—implicated in aging, carcinogenesis, and degenerative diseases—minimizing oxidative degradation during processing is critical (10, 11).

Despite these encouraging results, long-term studies assessing the safety, efficacy, and potential adverse effects of crocodilian oil in skin aging are lacking. Further clinical research is necessary to validate its dermatological applications and optimize its formulations for both cosmetic and therapeutic use.

### 5.2 Affects energy metabolism

Energy metabolism is a fundamental physiological process, with mitochondria playing a central role in maintaining cellular energy homeostasis. These organelles regulate metabolism through glycolysis, fatty acid oxidation, the tricarboxylic acid cycle, and oxidative phosphorylation, thereby meeting the energy demands essential for survival. In addition to energy production, mitochondria influence critical cellular functions, including differentiation, proliferation, and maintenance of homeostasis (43).

Crocodilian oil, rich in polyunsaturated fatty acids, has been shown to support mitochondrial function and enhance cerebral energy production, suggesting potential cognitive and neuroprotective benefits (44). In animal models, oral administration of crocodilian oil revealed no signs of acute toxicity, indicating its safety as a dietary supplement (16). Mechanistically, crocodilian oil modulates the expression of energy-regulating proteins through activation of the AMPK signaling pathway, thereby promoting hepatic energy metabolism and contributing to systemic energy balance (45). It also influences mitochondrial morphology by upregulating hydrolase domain-containing protein 3, a key factor in maintaining mitochondrial integrity (46). These findings suggest a potential role for crocodilian oil as a dietary intervention in metabolic disorders affecting the liver and brain.

However, some studies report adverse outcomes. Linn and Thiri Wai (47) found that crocodilian oil exacerbated renal injury in diabetic models by impairing mitochondrial homeostasis and promoting mitochondrial dysfunction. These effects may be linked to an imbalanced fatty acid composition—particularly a high omega-6 to omega-3 ratio and elevated palmitic acid content—highlighting the need for caution in its use among individuals with diabetic nephropathy.

Beyond mitochondrial function, crocodilian oil has also been linked to the modulation of gut microbiota. Parunyakul et al. demonstrated that supplementation increased the abundance of nitrogen-fixing spirochetes and ileal leptospires, suggesting that crocodilian oil may serve as an alternative fat source capable of supporting host metabolism and intestinal microbial balance (48). Given the interplay between gut microbiota and mitochondrial health, crocodilian oil may exert dual benefits by restoring mitochondrial energy metabolism and promoting microbial homeostasis (49). These findings underscore the need for further research into its potential in managing metabolic and gastrointestinal disorders.

In addition, crocodilian oil may help regulate blood lipids and reduce the risk of blood clots. Crocodilian oil can reduce triglycerides (48). Its atherosclerotic index is 0.29 and its thrombogenic index is 0.47 (13), both showing relatively low values. This indicates that its consumption does not pose a risk to the development of these diseases in the human body and promotes the prevention of the increasing occurrence of cardiovascular diseases (50).

### 5.3 Affects liver biotransformation

The liver plays a central role in detoxifying endogenous and exogenous compounds through biotransformation, a metabolic process that increases the water solubility of lipophilic substances to facilitate their excretion via bile or urine. Hepatic biotransformation occurs in two phases. Phase I reactions—primarily mediated by cytochrome P450 monooxygenases — involve oxidation, reduction, and hydrolysis. Phase II reactions entail conjugation processes that attach polar functional groups, such as glutathione, sulphate, or glucuronic acid, to Phase I metabolites, enhancing their elimination. Of these, glutathione conjugation, catalyzed by glutathione S-transferases (GSTs), serves as a key defense mechanism against electrophilic xenobiotics (51).

Pitchaya et al. (52) reported that crocodilian oil may support hepatic biotransformation even under high-fat dietary conditions by modulating the expression of detoxification enzymes. Specifically, supplementation with crocodilian oil significantly reduced cytochrome P450 1A2 activity and mRNA expression, along with decreased expression of the GSTA1 gene. These changes were associated with reduced lipid peroxidation in liver tissue suggesting a potential hepatoprotective effect. However, it remains unclear whether crocodilian oil affects other critical detoxification enzymes or pathways. Further studies are needed to investigate its influence on aryl hydrocarbon receptor–dependent signaling, especially in the context of diet-induced hepatic stress.

Currently, limited data exist regarding the pharmacokinetics of crocodilian oil, including its absorption, distribution, metabolism, and excretion in humans. Additionally, its long-term safety—particularly regarding hepatic and renal function—has not been fully established. Comprehensive pharmacological and toxicological evaluations are essential to determine its therapeutic potential and ensure its safe use in clinical settings.

### 5.4 Antioxidant activity

Antioxidant activity refers to the ability to neutralize ROS or inhibit their formation, thereby mitigating oxidative stress and maintaining redox homeostasis. Oxidative stress, resulting from an imbalance between free radical production and the body’s antioxidant defenses, is a major contributor to aging and the development of various diseases (53).

The antioxidant potential of monounsaturated and polyunsaturated fatty acids has been well documented. Srisuksai et al. (54) demonstrated that crocodilian oil exhibits significantly greater free radical scavenging activity than olive oil, particularly in detoxifying hydrogen peroxide. This effect is likely due to its high content of unsaturated fatty acids and antioxidant compounds such as vitamin E that stabilize free radicals and protect cellular components from oxidative damage (11, 42, 44). Crocodilian oil has also been shown to enhance the activity of SOD, a key endogenous antioxidant enzyme that catalyzes the dismutation of superoxide radicals into oxygen and hydrogen peroxide, further contributing to its antioxidative effects. In addition to its antioxidant capacity, crocodilian oil has demonstrated triglyceride-lowering effects, suggesting a dual role in modulating lipid metabolism and oxidative stress. These combined properties highlight its potential as a supplemental dietary oil for the prevention and management of conditions associated with oxidative damage and hypertriglyceridaemia (54).

## 6 Conclusion

### 6.1 Bioactive components and therapeutic potential of crocodilian oil

Crocodilian oil has garnered growing scientific interest due to its rich composition of bioactive compounds, particularly unsaturated fatty acids and stearic acid, which underlie its antimicrobial, anti-inflammatory, antioxidant, and skin-reparative properties. Stearic acid has demonstrated broad-spectrum antimicrobial activity against both Gram-positive and Gram-negative bacteria, including multidrug-resistant strains such as *Staphylococcus epidermidis* and *Enterococcus faecalis*. Meanwhile, omega-3 and omega-6 polyunsaturated fatty acids contribute significantly to the oil’s anti-inflammatory effects, which are critical for wound healing and maintaining skin barrier function. For example, omega-6 linoleic acid supports barrier integrity and prevents transepidermal water loss, while omega-9 oleic acid reduces inflammation via activation of SIRT1, a key regulator of oxidative stress. Crocodilian oil also exhibits potent antioxidant activity. Comparative studies have shown that its free radical scavenging capacity exceeds that of olive oil, largely due to its high content of unsaturated fatty acids and vitamin E, which protect cellular components from oxidative damage.

Additionally, crocodilian oil has been shown to lower serum triglyceride levels, indicating potential as a functional dietary oil for managing oxidative stress and hyperlipidaemia. Collectively, these properties support its application not only in cosmetic products—such as moisturizers, barrier-repair formulations, and anti-aging treatments—but also in therapeutic contexts addressing inflammation, wound healing, and metabolic disorders.

### 6.2 Mechanisms in wound healing and scar reduction

The wound-healing properties of crocodilian oil have been well-documented in preclinical studies, with strong evidence supporting its ability to accelerate tissue repair and reduce scar formation. These therapeutic effects are closely associated with modulation of key signaling pathways, including p38 MAPK, TGF-β1/Smad3, and AMPK, which regulate inflammation, antimicrobial responses, fibroblast activity, and collagen synthesis—critical processes in wound resolution and tissue remodeling.

Notably, reduced expression of TGF-β1 and Smad3 in animal burn models has been linked to decreased fibroblast activation and collagen deposition, thereby lowering the risk of hypertrophic scarring. Crocodilian oil has also been shown to enhance wound closure, suppress inflammatory cytokine production, and promote re-epithelialization in thermal injury models, further supporting its reparative effects.

Despite these promising outcomes, the precise molecular mechanisms underlying the wound-healing and scar-reducing activities of crocodilian oil remain incompletely defined. Further research is needed to elucidate how its bioactive components—particularly unsaturated fatty acids—influence immune cell function, including the roles of T cells, macrophages, and other components of the innate and adaptive immune systems. Moreover, while topical application has demonstrated rapid and sustained anti-inflammatory effects, the pharmacokinetics, systemic effects, and long-term outcomes of oral administration are largely uncharacterized. These aspects are particularly important for evaluating its potential in managing chronic wounds and inflammatory dermatoses.

Although crocodilian oil shows promise in modulating collagen synthesis and immune signaling for scar reduction, well-designed clinical trials are essential to confirm its efficacy across diverse wound types, determine optimal dosing regimens, and guide formulation strategies for therapeutic application. Beyond dermatological uses, crocodilian oil has traditionally been employed in regions such as Mexico, Africa, China, and Southeast Asia to treat conditions ranging from asthma and emphysema to cancer, burns, and inflammation. However, these ethnomedicinal applications remain largely anecdotal and lack rigorous scientific validation. Future studies should expand the investigation of crocodilian oil to other disease models, including cancer, cardiovascular, and metabolic disorders, to fully explore its therapeutic potential.
