# Supplementary material for: Recent advances in crocodilian oil research: bioactive components and potential therapeutic applications
Source: Front Med (Lausanne). 2025 Jun 18;12:1573925. doi: 10.3389/fmed.2025.1573925 (PMC12213835; doi:10.3389/fmed.2025.1573925)

Supplementary Material

# Supplementary Figures and Tables

## Supplementary Figures


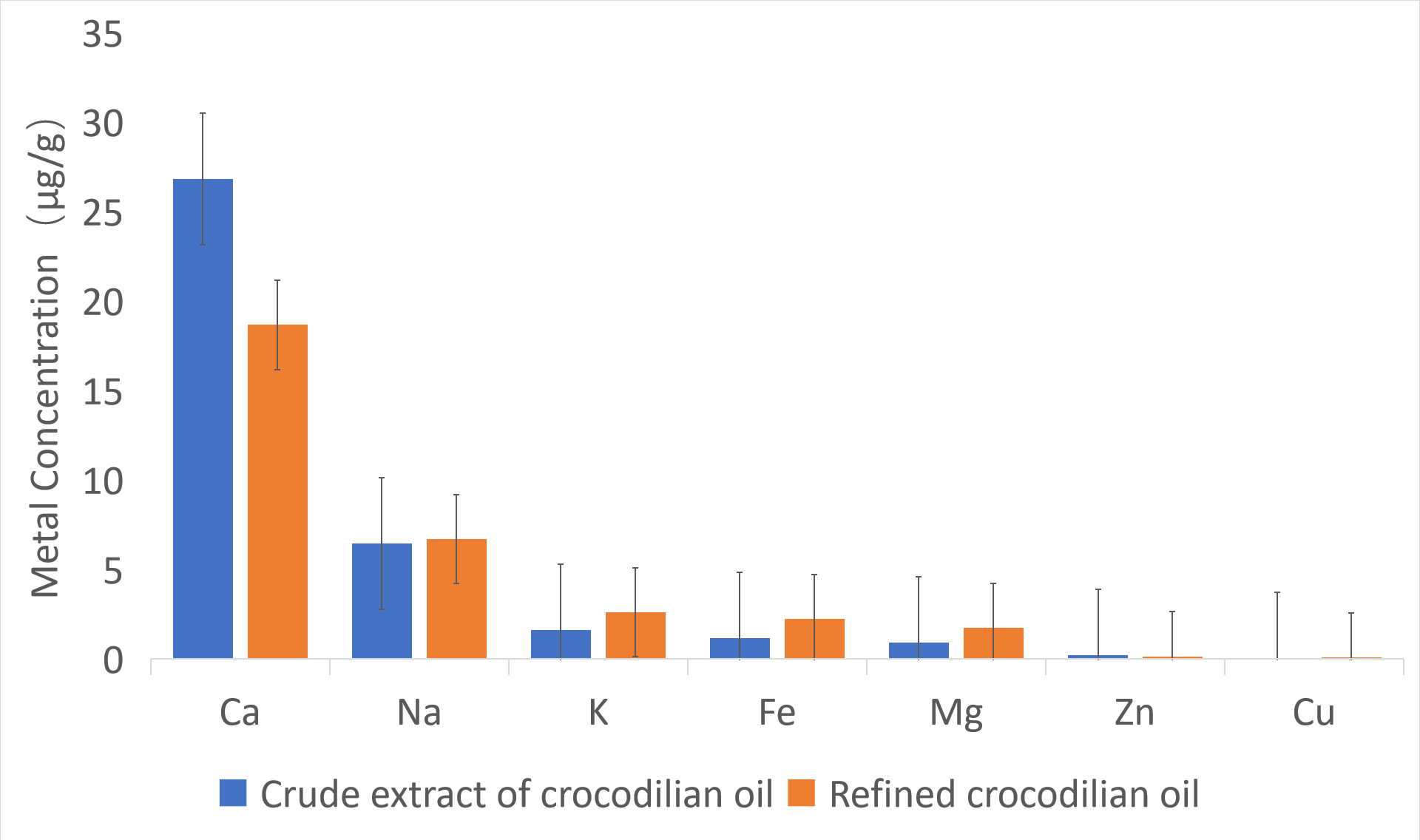


**Supplementary Figure 1. Concentration of metal elements in crocodilian oil**

The content of the macronutrient Ca is the highest both before and after the refining of crocodilian oil. The content of toxic elements in the refined alligator oil has significantly decreased and is all below the detection limit (<0.1 μg/g).

## Supplementary Tables

Supplementary Table 1


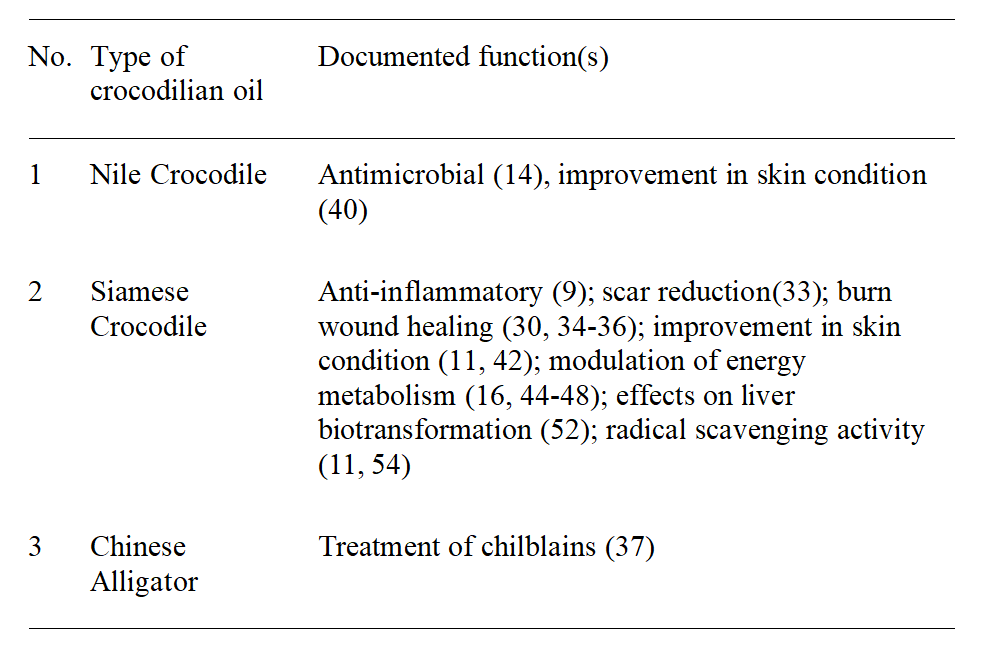

Supplement: Supplementary file 1 [file Supplementary_file_1.docx]
